# Frailty and pain in an acute private hospital: an observational point prevalence study

**DOI:** 10.1038/s41598-023-29933-x

**Published:** 2023-02-27

**Authors:** Rosemary Saunders, Kate Crookes, Karla Seaman, Seng Giap Marcus Ang, Caroline Bulsara, Max K. Bulsara, Beverley Ewens, Olivia Gallagher, Renée Graham, Karen Gullick, Sue Haydon, Jeff Hughes, Kim-Huong Nguyen, Bev O’Connell, Debra Scaini, Christopher Etherton-Beer

**Affiliations:** 1 Centre for Research in Aged Care, School of Nursing and Midwifery, Edith Cowan University, Joondalup, WA Australia; 2 School of Nursing and Midwifery, The University of Notre Dame Australia, Fremantle, WA Australia; 3 Institute for Health Research, The University of Notre Dame Australia, Fremantle, WA Australia; 4 Hollywood Private Hospital, Nedlands, WA Australia; 5 PainChek Ltd, Sydney, NSW Australia; 6 Curtin Medical School, Curtin University, Bentley, WA Australia; 7 Faculty of Medicine and Biomedical Sciences, The University of Queensland, Saint Lucia, QLD Australia; 8 Medical School, The University of Western Australia, Crawley, WA Australia; 9 Centre for Health Systems and Safety Research, Macquarie University, Sydney, Australia

**Keywords:** Diagnosis, Geriatrics, Health services

## Abstract

Frailty and pain in hospitalised patients are associated with adverse clinical outcomes. However, there is limited data on the associations between frailty and pain in this group of patients. Understanding the prevalence, distribution and interaction of frailty and pain in hospitals will help to determine the magnitude of this association and assist health care professionals to target interventions and develop resources to improve patient outcomes. This study reports the point prevalence concurrence of frailty and pain in adult patients in an acute hospital. A point prevalence, observational study of frailty and pain was conducted. All adult inpatients (excluding high dependency units) at an acute, private, 860-bed metropolitan hospital were eligible to participate. Frailty was assessed using the self-report modified Reported Edmonton Frail Scale. Current pain and worst pain in the last 24 h were self-reported using the standard 0–10 numeric rating scale. Pain scores were categorised by severity (none, mild, moderate, severe). Demographic and clinical information including admitting services (medical, mental health, rehabilitation, surgical) were collected. The STROBE checklist was followed. Data were collected from 251 participants (54.9% of eligible). The prevalence of frailty was 26.7%, prevalence of current pain was 68.1% and prevalence of pain in the last 24 h was 81.3%. After adjusting for age, sex, admitting service and pain severity, admitting services medical (AOR: 13.5 95% CI 5.7–32.8), mental health (AOR: 6.3, 95% CI 1. 9–20.9) and rehabilitation (AOR: 8.1, 95% CI 2.4–37.1) and moderate pain (AOR: 3.9, 95% CI 1. 6–9.8) were associated with increased frailty. The number of older patients identified in this study who were frail has implications for managing this group in a hospital setting. This indicates a need to focus on developing strategies including frailty assessment on admission, and the development of interventions to meet the care needs of these patients. The findings also highlight the need for increased pain assessment, particularly in those who are frail, for more effective pain management.

**Trial registration: **The study was prospectively registered (ACTRN12620000904976; 14th September 2020).

## Introduction

Increasing numbers of hospitalised older people present growing challenges for patient care, particularly given many of these patients are categorised as frail and have complex health care needs^1^. Frailty is defined as ‘a medical syndrome with multiple causes and contributors that is characterised by diminished strength, endurance and reduced physiological function, that increases an individual’s vulnerability for increased dependency and/or death’ [^2^, p393]

Frail patients in hospital are at increased risk of adverse events, including falls, clinical deterioration, delirium, post-operative complications, increased length of stay and higher levels of frailty, as well as death during hospitalisation^3–5^. The impact of frailty extends beyond the patient to the health care system due to increased care needs and management of frail older patients and the related costs^6^. Frail people often have multiple co-morbidities which adds another level of complexity to the management of this population^7^. Growing evidence suggests frailty and pain are closely linked^8,9^ and poor pain assessment and management can contribute to the progression of frailty^10–12^. A greater understanding of the prevalence of frailty in hospitals, and other associated factors such as pain, may result in improved care for frail patients and allow for the implementation of initiatives to reduce the progression of frailty^13^.

Prevalence studies of frailty in hospitals have often focussed on older adults^14,15^ or sub-groups of patients, for example surgical patients^16^, meaning that the prevalence and distribution of frailty across the entire hospital is not well understood. The paucity of studies in this area may be in part due to the challenges of administering many performance-based frailty assessment tools which often require lengthy assessments by specialists (e.g., phenotypic model^17^). The development of patient self-report frailty assessment tools, such as the Reported Edmonton Frail Scale (REFS)^18^ and the modified REFS (mod-REFS)^19^, which have been validated^18^, have enabled larger prevalence studies.

Richards et al.^20^ utilised the REFS to assess the point prevalence of frailty in a tertiary hospital in New Zealand. This study found the prevalence of frailty to be almost half of patients at 49%; frailty significantly increased with age and there were higher rates of frailty amongst patients admitted to a medical speciality. Similarly, Condon et al.^21^ found 52% of patients at an Irish tertiary hospital were frail. No previous studies have explored the point prevalence of both frailty and pain and their concurrence across an entire hospital.

The aim of this study was to determine the point prevalence of frailty and pain for all adult inpatients, excluding those in high dependency units, in an acute care private metropolitan hospital.

## Methods

### Patient population

The study was conducted at the largest private, acute care, metropolitan hospital in Western Australia with 860 licensed beds at the time of the study, providing care across surgical, medical, rehabilitation, mental health, critical care, and day procedure areas, but the hospital did not have an emergency department. Adult inpatients (aged over 18 years) admitted prior to 0800 h on one day in November 2020 were eligible to participate. Patients in day procedures, high dependency units (including the intensive care unit [ICU] and cardiac care unit [CCU]), patients with severe hearing impairment, severe intellectual disability and those considered too unwell to participate (as determined by the ward manager) were deemed ineligible and excluded.

Given the relationship between frailty and cognitive decline and, in order to fully capture frailty, it was important that cognitive decline was not an exclusion criterion. Processes were put in place to gain proxy consent from family members of patients with cognitive decline. Only two eligible patients were identified as requiring proxy consent due to cognitive decline but were unable to be recruited due to failure to contact the proxy.

### Data collection process

Data collectors worked in pairs with one pair assigned to each ward. They were either registered nurses, final-year nursing students, or allied health professionals. All data collectors received training on the data collection procedures and followed a data collection script. One member of each pair was a senior nurse from the assigned ward and was responsible for accessing patients’ medical records.

Prior to approaching patients, data collectors obtained a list of current patients and consulted with the ward nurse manager to identify any patients meeting the exclusion criteria. Patients were provided with a participant information sheet and a verbal description of the study. Patients were asked for verbal consent and their response was entered into a Qualtrics^22^ data collection tool running in the Qualtrics Offline Surveys application on an iPad or iPad-mini by the data collector. One data collector then performed the pain assessment followed by the frailty assessment while the other collected demographic and clinical information from the patient’s medical record.

### Frailty assessment

Frailty was assessed using the self-report modified Reported Edmonton Frail Scale (mod-REFS)^19^. This 13-item questionnaire evaluates multifunctional domains (cognition, general health, functional independence, social support, medication use, nutrition, mood and continence), and produces a score between 0 and 18 with scores of 8 and above classified as frail^19^. Scores were also coded according to severity of frailty: 0–5 not frail, 6–7 apparently vulnerable, 8–9 mild frailty, 10–11 moderate frailty and 12–18 severe frailty^19^. The data collector asked the patient the questions and recorded their responses in Qualtrics. To explore self-perceptions of frailty, participants were asked 1) Do you consider yourself to be frail? (yes/no);and 2) Based on your answer to the previous question, on a scale of 0–10 where 0 is ‘not at all frail’ and 10 is ‘extremely frail’, how frail do you think you are? Participants were instructed to respond to the questions based on their abilities prior to admission and their self-reported performance two weeks prior to admission^19^.

### Pain assessment

The participants were asked to rate the intensity of their current pain verbally using the standard numeric rating scale where 0 is no pain and 10 is the worst possible pain. They were then asked to rate the intensity of their worst pain experienced in the last 24 h from 0 to 10 and whether that was whilst resting or on movement. Pain ratings were recorded by the data collector in Qualtrics. For the prevalence analysis of both current pain and worst pain in the last 24 h, pain ratings of 0 were coded as no pain and ratings of 1–10 as pain reported. For modelling, ratings were coded according to severity of pain: 0 = no pain, 1–3 = mild pain, 4–6 = moderate pain, and 7–10 = severe pain^23^. To enable pain assessment in patients with cognitive decline, pain was assessed using the PainChek® application^24,25^. PainChek® is an evidence-based pain management system that utilises automated facial recognition and analysis to recognise facial action units indicative of pain presence, combined with user completed checklists of pain behaviour to calculate a pain intensity score (https://www.painchek.com/). Pain scores were recorded within the application on either on an iPad or iPad-mini.

### Demographic and clinical data

Demographic and clinical information relevant to frailty and pain were collected from the patient’s medical record. This information included year of birth, sex, admission type (elective/acute), indigenous status and pre-admission residential status (home/residential aged-care facility). Admitting diagnosis, active medical conditions, and prescribed analgesics (dosage and time of last dose) were also collected but are not reported here.

These data were collected on paper forms and then, after data collection, entered into a Qualtrics form by two members of the research team. Any missing information identified at the data cleaning stage of analysis was requested from the ward nurse manager who then retrieved the missing information from the patient’s medical record. Classification of admitting service (i.e., surgical/medical/rehabilitation/mental health) was performed post hoc by the administrative health information coding staff.

### Statistical analysis

All recruited participants were able to provide informed consent and self-report pain, therefore only the numeric rating scale pain data are reported. Categorical variables were reported as whole numbers and percentages and continuous as mean (standard deviation) and median (Interquartile ranges). Chi-square test was conducted for categorical variables and Kruskal Wallis for continuous variables. Spearman rank-order correlation coefficient tests were conducted to determine if there was a correlation between the mod-REFS score and self-reported ‘how frail do you think you are?’, and between current pain and worst pain in the last 24 h. Cohen’s kappa was used to measure the interrater agreement between the mod-REFS (frail or not frail) and ‘do you consider yourself frail’ (yes/no)^26^. Chi-square tests were used to determine if there was a difference in categorical variables between patients categorised as frail versus not frail according to the mod-REFS. Univariate and multivariate binary logistic regression was performed to identify factors predictive of frailty. Regression coefficients were reported as odds ratios with 95% confidence intervals. The area under the receiver-operating characteristic (ROC) curve was used to determine the goodness-of-fit for the multivariate model. The variables included in the final adjusted model included age, sex, admitting service and current pain. Admission type was not included due to collinearity with admitting service, similarly for pain in the last 24 h and current pain and for self-report frailty and mod-REFS score. Current pain as a continuous variable was highly skewed, current pain severity was therefore included as a categorical variable.

The standard level of statistically significance of 0.05 was applied. All analyses were performed using STATA SE (version 15)^27^. The reporting of this study complies with the Strengthening the Reporting of Observational Studies in Epidemiology Statement (STROBE) Checklist^28^ (Appendix S1).

### Ethical considerations

Ethical approval was granted by the Human Research Ethics Committees of Ramsay Health Care (WA/SA) (ref: 2038) and Edith Cowan University (ref: 2020-02008-SAUNDERS). All methods were performed in accordance with the relevant guidelines and regulations of the HREC approving institutions. A verbal consent process was approved by as the research was considered low risk, the interaction with the patient was minor, and assessments of pain and frailty are part of standard care, verbal consent from patients was sought and their response recorded by the data collector in Qualtrics. If a patient was unable to provide informed verbal consent due to cognitive impairment or inability to verbally communicate, written proxy consent would have been sought from their guardian or next-of-kin following guidelines from the Western Australian Department of Health to adhere to the requirements of the Western Australian Guardianship and Administration Amendment (Medical Research) Act 2020. However, there were no patients in the study sample who were unable to provide informed consent.

## Results

### Recruitment

**Figure 1:**
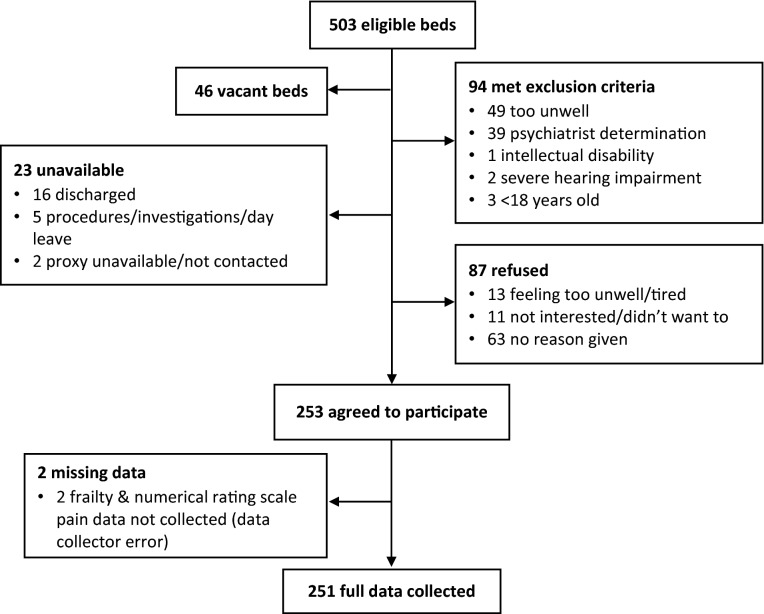
Participant recruitment flowchart.

At 0800 h on the day of data collection, 457 of the 503 eligible beds were occupied. Consent was obtained from 253 patients (55.4% of occupied eligible beds) and full data was collected from 251 patients (54.9%). Frailty and numeric rating scale pain data were not collected for two patients due to data collector error. Of the 204 excluded patients, 87 (19.0%) refused, 23 (5.0%) were unavailable and 94 (20.6%) met exclusion criteria (Fig. 1). Due to small sample size, the factors of indigeneity (Indigenous n = 1) and pre-admission residential status (residential aged-care facility n = 4) were not explored.

### Prevalence

Of the 251 participants with complete data, 67 (26.7%) were classified as frail according to the mod-REFS (Table 1). In terms of severity, 13.5% had mild frailty, 7.6% moderate frailty and 5.6% severe frailty. When asked if they considered themselves to be frail 66 participants (26.3%) responded ‘yes’. There was a significant positive correlation between mods-REF score and self-reported frailty rating, *r*s = 0.59, *p* < 0.001 (Spearman). The level of agreement between mod-REFS frailty status (frail/not frail) and self-reported ‘do you consider yourself to be frail?’ (yes/no) was 80.5%, however, Cohen’s kappa of 49.9% suggests this is a moderate strength of agreement between the two measures.

**Table 1:** Prevalence of frailty and pain.

	N (%)
Frailty	
mod-REFS	
Frailty status (mod-REFS score)	
Frail (8 or above)	67 (26.7%)
Not frail (0–7)	184 (73.3%)
Frailty severity (mod-REFS score)	
Severe frailty (12–18)	14 (5.6%)
Moderate frailty (10–11)	19 (7.6%)
Mild frailty (8–9)	34 (13.6%)
Apparently vulnerable (6–7)	36 (14.3%)
Not frail (0–5)	148 (59.0%)
Self-report	
Do you consider yourself to be frail?	
Yes	66 (26.3%)
No	185 (73.7%)
Pain	
Current pain (pain rating)	
Pain (1–10)	171 (68.1%)
No pain (0)	80 (31.9%)
Pain severity (pain rating)	
Severe pain (7–10)	13 (5.2%)
Moderate pain (4–6)	55 (21.9%)
Mild pain (1–3)	103 (41.0%)
No pain (0)	80 (31.9%)
Worst pain in last 24 h (pain rating)	
Pain (1–10)	204 (81.3%)
No pain (0)	47 (18.7%)
Pain severity (pain rating)	
Severe Pain (7–10)	94 (37.5%)
Moderate pain (4–6)	72 (28.7%)
Mild pain (1–3)	38 (15.1%)
No pain (0)	47 (18.7%)

*mod-REFS* modified Reported Edmonton Frail Scale.

Current pain was reported by 68.1% of participants and 81.3% of participants reported pain in the last 24 h (Table 1). There was a strong positive correlation between ratings of current pain and pain in the last 24 h that was statistically significant, *r*s = 0.63, *p* < 0.001 (Spearman).

Patient characteristics by mod-REFS frailty status are reported in Table 2. Frail patients were more likely to be older, admitted as an acute admission, to a medical specialty and in more pain than patients who were not frail. There was no difference observed between sex. Supplementary Table 1A, demonstrated the mod-REFS frailty scores by admitting service.

**Table 2:** Patient characteristics by frailty type.

Characteristics	Total (n = 253)	Not Frail (n = 184)	Frail (n = 67)	p value*
Age,				
Mean (SD)	**63.26 (17.41)**	**61.70 (17.06)**	**68.15 (17.51)**	**0.004**
Median (IQR)	**66 (55–75)**	**65 (54–73)**	**72 (56–81)**	
Age, n(%)				**0.007**
< 65	112	89 (79%)	23 (21%)	
65–69	34	28 (82%)	6 (18%)	
70–74	39	28 (72%)	11 (28%)	
75–79	27	20 (74%)	7 (26%)	
80–84	19	10 (53%)	9 (47%)	
85 +	20	9 (45%)	11 (55%)	
Sex, n (%)				0.901
Female	124 (49.0%)	89 (48.4%)	33 (49.2%)	
Male	129 (51.0%)	95 (51.6%)	34 (50.8%)	
Admission type, n (%)				**0.003**
Acute	97 (38.3%)	61 (33.2%)	36 (53.7%)	
Elective	156 (61.7%)	123 (66.8%)	31 (46.3%)	
Admitting service, n (%)				**0.000**
Medical	75 (29.6%)	34 (18.5%)	41 (61.2%)	
Mental Health	28 (11.1%)	20 (10.9%)	8 (11.9%)	
Rehabilitation	21 (8.3%)	12 (6.5%)	9 (13.4%)	
Surgical	129 (51.0%)	118 (64.1%)	9 (13.4%)	
Current pain, n (%)				**0.000**
No pain	80 (31.9%)	55 (29.9%)	25 (37.3%)	
Mild pain	103 (41.0%)	89 (48.4%)	14 (20.9%)	
Moderate pain	55 (21.9%)	34 (18.5%)	21 (31.3%)	
Severe pain	13 (5.2%)	6 (3.3%)	7 (10.4%)	

*Chi-square tests. Bold means statically significant.

Table 3 outlines the unadjusted and adjusted odds ratios for frailty. The adjusted model demonstrated that medical (AOR: 13.5 95% CI 5.7–32.8), rehabilitation (AOR: 8.1, 95% CI 2.4–37.1) and mental health patients (AOR: 6.3, 95% CI 1.9–20.9) were all more likely to be frail than surgical patients. Additionally, those in moderate pain were more likely to be frail compared to those reporting mild pain (AOR: 3.90, 95% CI 1.6–9.8). The adjusted model had an area under ROC curve of 0.84, which indicates excellent fit in identifying frail patients.

**Table 3:** Unadjusted and adjusted odds ratios for frailty.

	Unadjusted OR (95%CI)	Adjusted OR (95%CI)
Age		
< 65	1 (Base)	1 (Base)
65–69	0.83 (0.31–2.24)	0.81 (0.23–2.78)
70–74	1.52 (0.66- 3.50)	1.47 (0.51–4.23)
75–79	1.35 (0.51–3.59)	0.105 (0.31–3.59)
80–84	3.48 (1.27–9.57)	3.26 (0.85–12.56)
85 +	4.73 (1.75–12.77)	2.20 (0.61–7.86)
Sex		
Female	1 (Base)	1 (Base)
Male	0.97 (0.55–1.69)	0.86 (0.42–1.76)
Admitting service		
Medical	15.81 (6.99–35.76)	13.65 (5.69–32.79)
Mental Health	5.24 (1.81–15.19)	6.28 (1.89–20.86)
Rehabilitation	9.83 (3.28–29.49)	8.06 (2.40–37.08)
Surgical	1 (Base)	1 (Base)
Current pain		
No pain	2.89 (1.38–6.03)	1.66 (0.71–3.88)
Mild pain	1 (Base)	1 (Base)
Moderate pain	3.93 (1.79–8.59)	3.90 (1.56–9.78)
Severe pain	7.41 (2.17–25.31)	4.00 (0.91–17.62)

Note: Adjusted for age, sex, admitting service and current pain.

## Discussion

The findings of this study indicate the importance of understanding the prevalence of both frailty and pain in hospitalised patients and add to the growing evidence of a relationship between frailty and pain.

Overall, the point prevalence of frailty in the hospital (26.7%) was lower than a previous hospital-wide study (that was conducted at a similar sized hospital and also used a self-report frailty scale) which found 49% of patients were frail^20^. However, the previous study was conducted in a public hospital whereas the current study was conducted in a private hospital. There is key socio-economic differences between patients admitted to public and private hospitals, and although not measured in this study, it may have contributed to the observed difference in frailty prevalence rate. The higher proportion of surgical patients (51.0%) in the current study compared to a previous study (38.1%)^20^ is likely to have contributed to the overall prevalence of frailty in the current study. As in previous studies, surgical patients were less likely to be frail than medical patients. Given the majority (78%) were undergoing elective surgery, frailty prevalence might have been be influenced by the pre-operative assessment of potential patients for eligibility for surgery at a private hospital.

Consistent with previous studies, this study found older patients were more likely to be frail, however age was not a significant predictor of frailty in the adjusted model. Frailty was not restricted to older adults with 34% of frail patients being under the age of 65. This finding combined with the high prevalence of frailty, particularly in medical (55%) and rehabilitation (43%) patients, highlights the potential value of assessing all patients to identify those who are frail and direct appropriate care and interventions during their inpatient stay and on discharge. Assessment of frailty is potentially not routinely completed in the acute care setting partially due the time and expertise required to administer some assessment tools^29,30^. The mod-REFS self-report questionnaire used in this study may be useful for this purpose as it is a quick self-assessment taking approximately 5 min, can be administered by nurses and a version of this questionnaire (REFS^18^) has been validated for use in hospitals^18^. Alternatively, the development of comprehensive admission assessments such as those developed by InterRAI that automatically include frailty measures along with other standard risk assessments may prove valuable in the future to inform care interventions and reduce administration at admission^31^.

The prevalence of frailty in the mental health wards (28.6%) was relatively high particularly given the relatively young age of these participants (median 35.5 years). The mod-REFS has not been validated with this population so these results may not reflect physical frailty and should be interpreted with caution. There is limited research in this area particularly with younger mental health patients. A previous study found the prevalence of frailty in older adults (mean age 74.6 years) in an acute psychiatric hospital was 37.9%^32^. Frailty in mental health patients is an important area for future research.

Overall, pain was highly prevalent across the participants both when reporting current pain (68.1%) and worst pain level in the last 24 h. Of those participants (81.3%) who reported pain in the last 24 h, 37.5% rated their worst pain as severe. Comparison with other studies is difficult due to variation in methodologies and patient cohorts, however, these results are similar to previous studies that have found the point prevalence of current pain in hospitals to be between 37.7% and 84.0% and worst pain in the last 24 h to be between 52 and 65%^33^. Given the distress that pain can cause patients, effective pain assessment and pain management should be prioritised in hospitals and more education provided to staff. Our finding in this cohort study that higher levels of pain predicted frailty supports recent findings of an association between frailty and pain^8,9^.

There are some limitations to the current study. First, there are inherent issues with the use of self-report measures within a health setting due to either over or under reporting. Furthermore, the self-report measure of frailty may not have accurately assessed frailty, as it is possible that a physical performance-based measures of frailty^17^ (including walking speed and hand grip strength) may have produced different estimates of prevalence. The lack of consensus on how best to assess and measure frailty is a barrier to comparison across studies^34^. Second, the results of this study may also be limited in their generalisability. Given that private hospitals account for approximately 40% of all hospital admissions in [redacted for blind review]^35^ it is important to understand the prevalence and distribution of frailty in that setting. While the patient population is likely comparable to other [redacted for blind review] private hospitals without emergency departments, those with emergency departments may have different prevalence and distribution of frailty. For example, there might be a higher number of frail surgical patients admitted acutely through emergency requiring surgical interventions following trauma. Thirdly neither the mod-REFS^19^ nor the REFS have been validated in the younger age group, but the REFS has been utilised in another hospital wide prevalence study^20^.

## Conclusion

This study adds to the growing evidence that frailty is prevalent in hospital patients, particularly older medical patients. Given that the majority of patients in this study, who were identified as being frail were aged over 65 years, managing this group of patients to improve health outcomes needs some attention. This finding highlights the importance of routine assessment of frailty on admission to direct appropriate care. The findings also argue for the development and implementation of interventions directed at this large population of vulnerable patients. The high prevalence of pain experienced by patients in this study, and the association with frailty, identifies the need for more consistent and routine pain assessment and management to improve patient outcomes. Therefore, assessment and management of both pain and frailty are vital to improve patient care and tailor discharge advice.

## Supplementary Information

Supplementary Information 1.

Supplementary Information 2.

## Data Availability

The datasets analysed during the current study are available from the corresponding author on reasonable request.
