# Supplementary material for: Frailty and pain in an acute private hospital: an observational point prevalence study
Source: Sci Rep. 2023 Feb 27;13:3345. doi: 10.1038/s41598-023-29933-x (PMC9971208; doi:10.1038/s41598-023-29933-x)
Supplement: Supplementary file 2 — Supplementary Information 2. [file 41598_2023_29933_MOESM2_ESM.docx]

Supplementary Material:

Table 1A. Frailty mod-REFs score by admitting service

|  |  | Admitting service | | | |
| --- | --- | --- | --- | --- | --- |
| **Frailty severity (mod-REFS score)** | Overall | Medical (n=75) | Mental Health (n=28) | Rehabilitation (n=21) | Surgical (n=127) |
| Severe frailty (12-18) | 14 (5.6%) | 10 (13.3%) | 1 (3.6%) | 3 (14.3%) | 0 |
| Moderate frailty (10-11) | 19 (7.6%) | 13 (17.3%) | 2 (7.1%) | 2 (9.5%) | 2 (1.6%) |
| Mild frailty (8-9) | 34 (13.6%) | 18 (24.0%) | 5 (17.9%) | 4 (19.1%) | 7 (5.5%) |
| Apparently vulnerable (6-7) | 36 (14.3%) | 7 (9.3%) | 6 (21.4%) | 7 (33.3%) | 16 (12.6%) |
| Not frail (0-5) | 148 (59.0%) | 27 (36.0%) | 14 (50.0%) | 5 (23.8%) | 102 (80.3%) |
